# Synovial Tissue Biopsy Research

**DOI:** 10.3389/fmed.2019.00072

**Published:** 2019-04-16

**Authors:** Douglas J. Veale

**Affiliations:** The Centre for Arthritis and Rheumatic Disease, University College Dublin, St. Vincent's University Hospital, Dublin, Ireland

**Keywords:** synovial tissue biopsy, rheumatoid arthritis, single cell analysis, biomarkers, synovial tissue biomarkers

## Abstract

Synovial tissue is a key structure in diarthrodial joints and is the primary target of inflammation in autoimmune arthritis. The study of synovial tissue has developed significantly in the last two decades as arthroscopic and ultrasonographic techniques have allowed visualization and access to synovial biopsy. Further progress in synovial tissue processing and analysis has improved studies of disease pathogenesis, biomarker discovery, and molecular therapeutic targeting with increasingly specialized analytical and technological approaches. In September 2018 the first course on Synovial Tissue Biopsies was convened in Brussels, in this Mini Review these approaches will be described and I will summarize how synovial tissue research advanced.

## Key points

- Synovial tissue is the target tissue of rheumatoid arthritis (RA).
- Synovial biopsy, under local anesthetic, is safe and well-tolerated by patients
- Cellular and molecular analysis of the synovial tissue of RA patients might identify novel targets for therapy and specific biomarkers.
- Technological advances in single cell and molecular analysis provides new opportunities for discovery.

## Introduction and History

The main focus of synovial tissue research has been rheumatoid arthritis (RA), as the most prevalent cause of inflammatory synovitis. In the last two decades, considerable advances have been made in the diagnosis and therapy of RA (1). However, early diagnosis and precision medicine remain a challenge. In the 1970's Ralph Schumacher and Barry Bresnihan pioneered synovial biopsy research using the Parker-Pearson needle to obtain biopsies and study the cellular composition of the tissue.

## Synovial Joint

**Figure 1:**
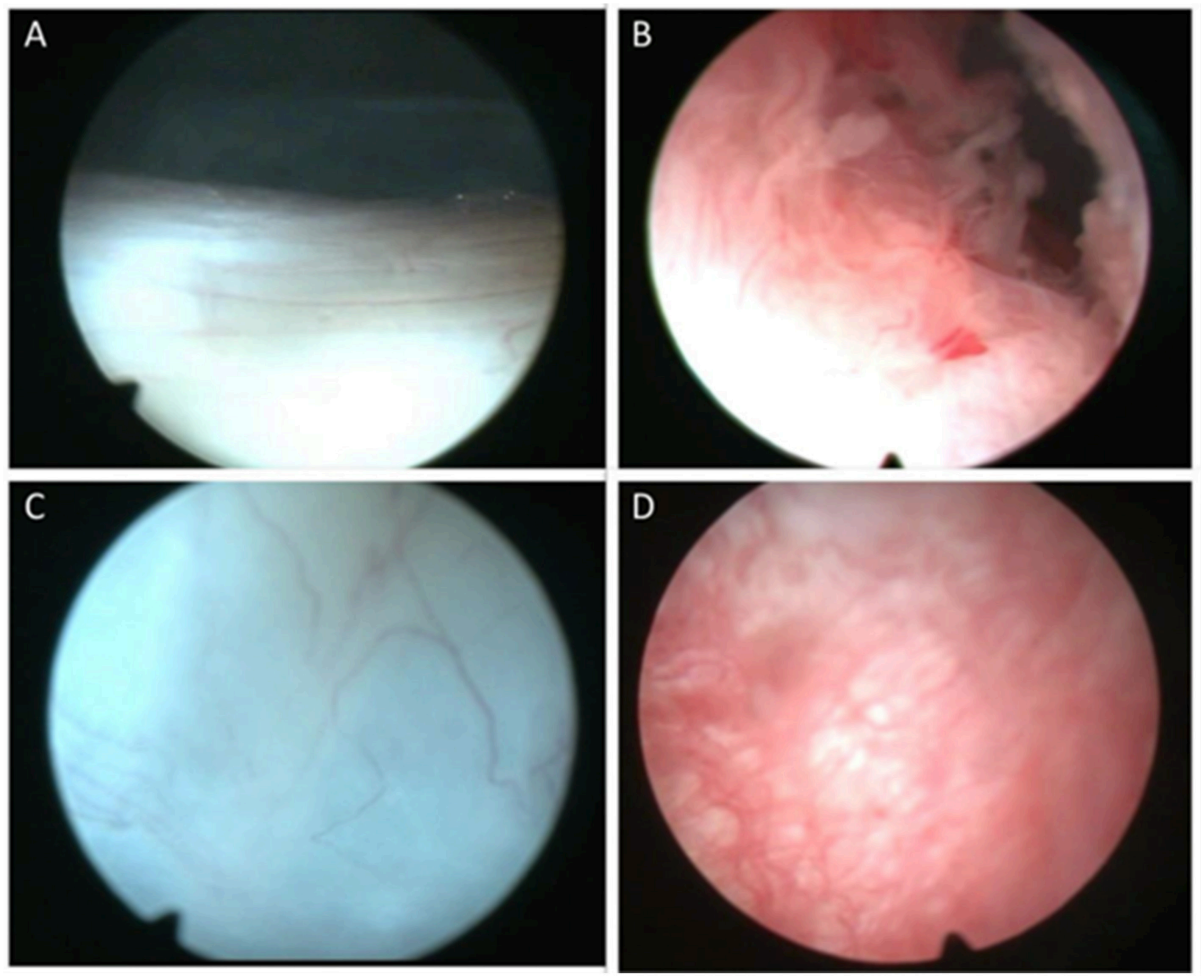
Representative macroscopic appearance of synovial tissue. Macroscopic images of the synovial tissue demonstrating normal synovial tissue **(A,C)** compared to inflamed and hyperplastic synovial villi in rheumatoid arthritis **(B,D)**.

Normal synovial tissue contains specialized fibroblast-like synoviocytes (FLS) interspersed with macrophages (2). In RA the synovial tissue becomes hypervascular and hyperplastic (Figure 1) while microscopic analysis reveals hyperplasia of the intimal lining layer, primarily due to increased accumulation of FLS and macrophage cells in the synovial lining (3). Angiogenesis, the development of new blood vessels is probably an early event enabling infiltration of immune cells such as T cells, B cells and monocytes, and is aberrant resulting in an abnormal blood vessel pattern (4). The new blood vessels appear immature and permit increased leukocyte migration, transforming the synovial tissue into an aggressive “pannus” characterized by release of proinflammatory cytokines from macrophages, T and B cells that stimulate FLS activation and subsequent cartilage and bone destruction (5–7). Although angiogenesis leads to increased blood vessels the tissue is markedly hypoxic *in vivo* (8).

## Synovial Biopsy

The analysis of synovial tissue biopsies has advanced our understanding of RA pathogenesis, yielded potential therapeutic targets, and allows detailed evaluation of new therapies (9, 10).

Synovial tissue biopsies have been obtained by blind needle biopsy, arthroplasty, arthroscopy, and more recently using ultrasound- (11). Arthroscopic and ultrasound-guided (USG) biopsy procedures are safe and well-tolerate, both provide good biopsy material. The main benefit of USG appears to be access to small joints, however the yield of synovial tissue is often lower (~80%) (12, 13), while in the authors experience arthroscopy provides 100% synovial tissue yields. There is a low adverse event rates of 0.9% for haemarthrosis, 0.2% for deep vein thrombosis, and 0.1% for both wound infection and joint infection (14). Similarly, a systematic review reported an overall major complication rate of 0.4% for ultrasound-guided biopsy procedures (15).

## Synovial Tissue Analysis

**Figure 2:**
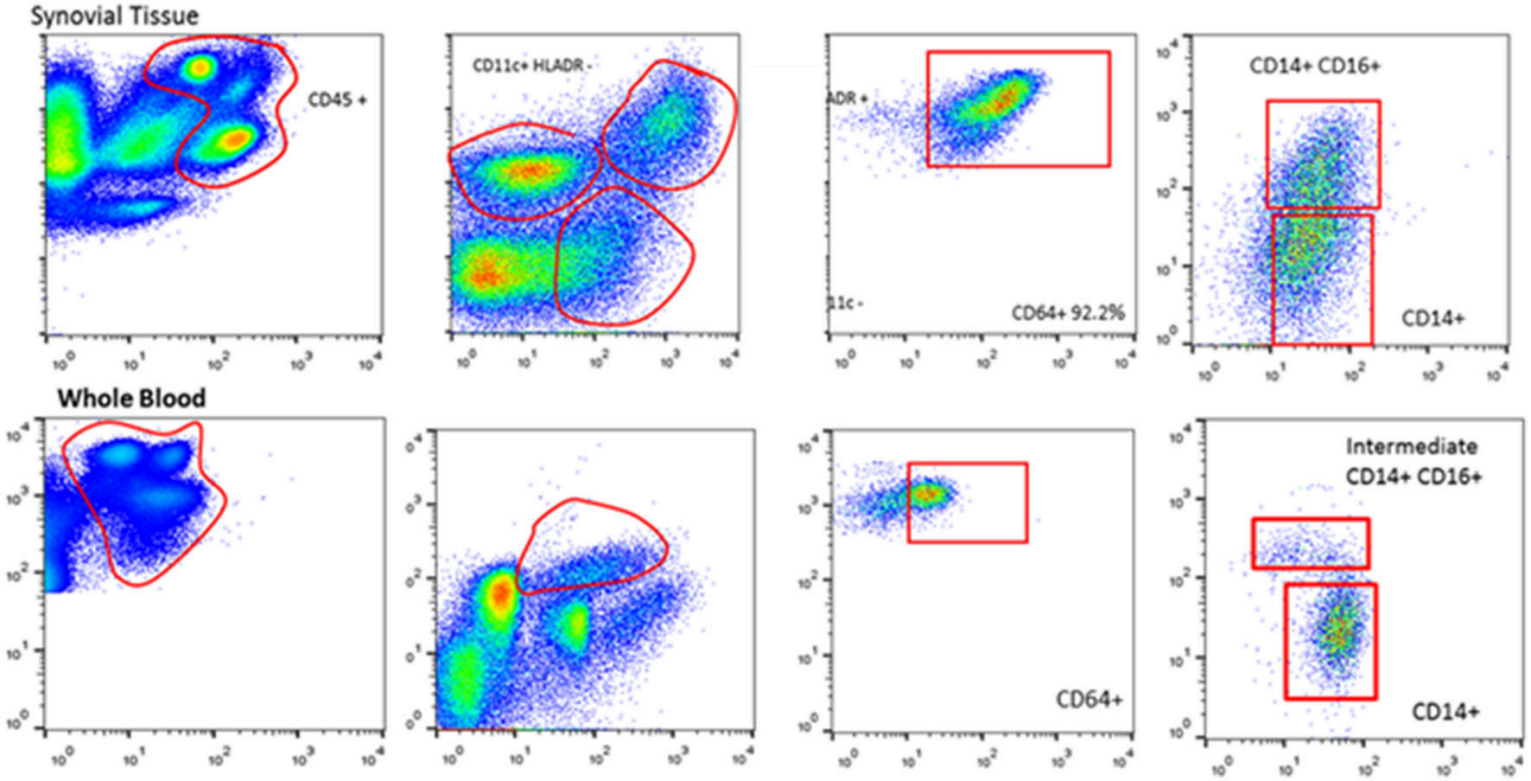
Synovial tissue and whole blood cell isolations with flow cytometry. Flow cytometric analysis of paired peripheral blood and synovial tissue derived cells illustrating the ability to isolate and identify single cell populations of key immune cells.

Immunohistochemical analysis of synovial tissue has a clinical role in the differential diagnosis of arthritis (e.g., infectious, granulomatous, infiltrative, or crystal arthropathies), the benefit in studies of personalized medicine have yet to provide a substantial advance (16). Interestingly though, studies of the synovium beyond immunohistochemistry involving whole-tissue culture, tissue digestion, homogenization, and single cell analysis with detailed molecular profiling including -omic technologies are now possible (Figure 2). Direct analysis of synovial tissue—the target of inflammation in RA—is critical to the investigation of pathogenesis in RA. Monocytes, T and B cells are expanded in the blood as well as in the synovial tissue of RA patients and this has provided the rationale for development of novel biological therapies including anti-cytokine antibodies, abatacept, and rituximab.

## Predictors Of Arthritis and Response to Therapy

In the last 15 years, synovial tissue analysis has impacted the treatment of early RA using clinical, pathological, and -omic data analysis. A link between circulating ACPAs and the development of RA in subjects with arthralgia, and bone damage has been described in patients with early arthritis (17, 18). The predictive value of a positive ACPA status in RA patients has been reported, however, in those with arthralgia it is highly variable with 30–70% subsequently developing RA on follow-up (19).One study has identified a highly expanded, T cell clone in RA synovial tissue early in the disease underlining the importance of T cells at this stage (20). Epigenetic changes in synovial tissue FLS might also define the different stages of RA after clinical onset (21).

Inflammatory genes overexpressed in pre-treatment biopsies might predict those RA patients most likely to responded to TNF inhibitor therapy. Another study of synovial tissue RNA suggested that transcripts associated with lymphocyte aggregates predicted response to infliximab therapy (22). The role of macrophages and T cells as biomarkers of response is also supported by gene-expression analyses of paired RA synovial biopsies before and after rituximab treatment, that showed clinical response was greater in those with high expression of macrophage and T cell associated synovial genes (23).

## Challenges in Biomarker Discovery

Recent advances in -omic techniques are allowing deeper molecular analysis of synovial tissue, however several challenges remain. The new technologies have become faster, better value and provide a more detailed analyses of genes, proteins, and epigenetic modifications. However, a number of commonly used microarray platforms have yielded poor reproducibility causing some problems with interpretation of data. In addition, the results of initial whole tissue transcriptional profiling await more detailed analysis. Therefore, in the last 2 years, we have developed laboratory techniques to dissociate the synovial biopsies into viable single cell subsets allowing specific analysis of the genes, proteins, and functions of the cell subsets that comprise the population in the actively inflamed synovial joint tissue (24).

## Conclusions

RA is characterized by inflammation of the synovial tissue, which therefore represents the target tissue of autoimmune arthritis. Various methods of sampling synovial tissue have now been validated as safe, well-tolerated by patients and minimally invasive thus they have become more widely practiced. In this Mini review I have focused on the development of synovial tissue biopsy studies including the current technological advances in analysis that allow detailed cellular and molecular experiments that define the functions of immune cells in the RA synovial tissue. These studies might allow greater understanding of the pathogenesis of RA and development of a “precision medicine” approach with improved therapy, patient stratification, development of new therapeutic targets, and development of specific biomarkers of response.

## Author Contributions

The author confirms being the sole contributor of this work and has approved it for publication.

### Conflict of Interest Statement

The author declares that the research was conducted in the absence of any commercial or financial relationships that could be construed as a potential conflict of interest.

## References

[1] Orr C Vieira-Sousa E Boyle DL Buch MH Buckley CD Cañete JD Synovial tissue research: a state-of-the-art review. Nat Rev Rheumatol. (2017) 13:463–75. 10.1038/nrrheum.2017.115 28701760

[2] Smith MD Barg E Weedon H Papengelis V Smeets T Tak PP. Microarchitecture and protective mechanisms in synovial tissue from clinically and arthroscopically normal knee joints. Ann Rheum Dis. (2003) 62:303–7. 10.1136/ard.62.4.303 12634226 PMC1754505

[3] Tak PP Smeets TJ Daha MR Kluin PM Meijers KA Brand R. Analysis of the synovial cell infiltrate in early rheumatoid synovial tissue in relation to local disease activity. Arthritis Rheum. (1997) 40:217–25. 10.1002/art.1780400206 9041933

[4] Reece RJ Canete JD Parsons WJ Emery P Veale DJ. Distinct vascular patterns of early synovitis in psoriatic, reactive, and rheumatoid arthritis. Arthritis Rheum. (1999) 42:1481–4. 10.1002/1529-0131(199907)42:7<1481::AID-ANR23>3.0.CO;2-E 10403277

[5] Ng CT Biniecka M Kennedy A McCormick J Fitzgerald O Bresnihan B. Synovial tissue hypoxia and inflammation *in vivo*. Ann Rheum Dis. (2010) 69:1389–95. 10.1136/ard.2009.119776 20439288 PMC2946116

[6] Mullan RH Matthews C Bresnihan B FitzGerald O King L Poole AR. Collagen biomarkers predict radiographic progression at one year in inflammatory arthritis patients after biologic therapy. Arthritis Rheum. (2007) 56:2919–28. 10.1002/art.22843 17763421

[7] Månsson B Carey D Alini M Ionescu M Rosenberg LC Poole AR. Cartilage and bone metabolism in rheumatoid arthritis. Differences between rapid and slow progression of disease identified by serum markers of cartilage metabolism. J Clin Invest. (1995) 95:1071–7. 10.1172/JCI117753 7533784 PMC441442

[8] Biniecka M Kennedy A Fearon U Ng CT Veale DJ O'Sullivan JN. Oxidative damage in synovial tissue is associated with in vivo hypoxic status in the arthritic joint. Ann Rheum Dis. (2010) 69:1172–8. 10.1136/ard.2009.111211 19706618

[9] Rooney M Whelan A Feighery C Bresnihan B. Changes in lymphocyte infiltration of the synovial membrane and the clinical course of rheumatoid arthritis. Arthritis Rheum. (1989) 32:361–9. 10.1002/anr.1780320402 2468336

[10] Firestein GS Paine MM Boyle DL. Mechanisms of methotrexate action in rheumatoid arthritis. Arthritis Rheum. (1994) 37:193–200. 10.1002/art.1780370207 8129774

[11] Gerlag DM Tak PP. How to perform and analyse synovial biopsies. Best Pract Res Clin Rheumatol. (2009) 23:221–32. 10.1016/j.berh.2009.01.006 19393567

[12] Kelly S Humby F Filer A Ng N Di Cicco M Hands RE. Ultrasound-guided synovial biopsy: a safe, well-tolerated and reliable technique for obtaining high-quality synovial tissue from both large and small joints in early arthritis patients. Ann Rheum Dis. (2015) 74:611–7. 10.1136/annrheumdis-2013-204603 24336336

[13] Najm A Orr C Heymann MF Bart G Veale DJ Le Goff B. Success rate and utility of ultrasound-guided synovial biopsies in clinical practice. J Rheumatol. (2016) 43:2113–9. 10.3899/jrheum.151441 27744399

[14] Kane D Veale DJ FitzGerald O Reece R. Survey of arthroscopy performed by rheumatologists. Rheumatology. (2002) 41:210–5. 10.1093/rheumatology/41.2.210 11886972

[15] Lazarou I D'Agostino MA Naredo E Humby F Filer A Kelly SG. Ultrasound-guided synovial biopsy: a systematic review according to the OMERACT filter and recommendations for minimal reporting standards in clinical studies. Rheumatology. (2015) 54:1867–75. 10.1093/rheumatology/kev128 26022188

[16] de Hair MJ Harty LC Gerlag DM Pitzalis C Veale DJ Tak PP. Synovial tissue analysis for the discovery of diagnostic and prognostic biomarkers in patients with early arthritis. J Rheumatol. (2011) 38:2068–72. 10.3899/jrheum.110426 21885519

[17] Bos WH Wolbink GJ Boers M Tijhuis GJ de Vries N van der Horst-Bruinsma IE. Arthritis development in patients with arthralgia is strongly associated with anti-citrullinated protein antibody status: a prospective cohort study. Ann Rheum Dis. (2010) 69:490–4. 10.1136/ard.2008.105759 19363023

[18] Nielen MM van der Horst AR van Schaardenburg D van der Horst-Bruinsma IE van de Stadt RJ Aarden L. Antibodies to citrullinated human fibrinogen (ACF) have diagnostic and prognostic value in early arthritis. Ann Rheum Dis. (2005) 64:1199–204. 10.1136/ard.2004.029389 15640269 PMC1755615

[19] Orr C Najm A Biniecka M McGarry T Ng CT Young F Synovial immunophenotype and anti-citrullinated peptide antibodies in rheumatoid arthritis patients: relationship to treatment response and radiologic prognosis. Arthritis Rheumatol. (2017) 11:2114–23. 10.1002/art.40218 28732135

[20] Klarenbeek PL de Hair MJ Doorenspleet ME van Schaik BD Esveldt RE van de Sande MG. Inflamed target tissue provides a specific niche for highly expanded T-cell clones in early human autoimmune disease. Ann Rheum Dis. (2012) 71:1088–93. 10.1136/annrheumdis-2011-200612 22294635

[21] Whitaker JW Shoemaker R Boyle DL Hillman J Anderson D Wang W. An imprinted rheumatoid arthritis methylome signature reflects pathogenic phenotype. Genome Med. (2013) 5:40. 10.1186/gm444 23631487 PMC3706831

[22] Lindberg J Wijbrandts CA van Baarsen LG Nader G Klareskog L Catrina A. The gene expression profile in the synovium as a predictor of the clinical response to infliximab treatment in rheumatoid arthritis. PLoS ONE. (2010) 5:e11310. 10.1371/journal.pone.0011310 20593016 PMC2892481

[23] Gutierrez-Roelens I Galant C Theate I Lories RJ Durez P Nzeusseu-Toukap A. Rituximab treatment induces the expression of genes involved in healing processes in the rheumatoid arthritis synovium. Arthritis Rheum. (2011) 63:1246–54. 10.1002/art.30292 21337318

[24] Canavan M Walsh AM Bhargava V Wade SM McGarry T Marzaioli V. Enriched Cd141+ DCs in the joint are transcriptionally distinct, activated, and contribute to joint pathogenesis. JCI Insight. (2018) 3:95228. 10.1172/jci.insight.95228 30518680 PMC6328029

